# High-throughput metabolic engineering of *Yarrowia lipolytica* through gene expression tuning

**DOI:** 10.1073/pnas.2426686122

**Published:** 2025-06-03

**Authors:** Wei Jiang, Shengbao Wang, Daniel Ahlheit, Tommaso Fumagalli, Zhijie Yang, Shreemaya Ramanathan, Xinglin Jiang, Tilmann Weber, Jonathan Dahlin, Irina Borodina

**Affiliations:** ^a^The Novo Nordisk Foundation Center for Biosustainability, Technical University of Denmark, Kgs. Lyngby DK-2800, Denmark; ^b^Department of Sustainable Biotechnology, Aalborg University, Aalborg East DK-9220, Denmark; ^c^Department of Biotechnology and Biosciences, University of Milano-Bicocca, Milano 20126, Italy

**Keywords:** high-throughput genome editing, CRISPR-Cas9, transcription factors, functional genomics, industrial biotechnology

## Abstract

The challenge of accurately predicting which genetic alternations lead to the desired phenotype necessitates high-throughput metabolic engineering approaches where numerous hypotheses can be tested simultaneously. We describe the CRISPR-Cas9-based method TUNE^YALI^ that enables high-throughput tuning of gene expression in the common industrial yeast *Yarrowia lipolytica*. The method is based on replacing the promoters of the target genes with native *Y. lipolytica* promoters of varying strengths or removing the promoters entirely. To demonstrate the method’s capabilities, we created a plasmid library that targets 56 transcription factors (TFs) and changes the expression of each TF to seven different levels. We transformed this library into reference and betanin-producing strains of *Y. lipolytica* and screened the resulting clones for changes in morphology, thermotolerance, or improved betanin production. The genetic markup of the yeast clones with the desired phenotypic changes was determined by sequencing the inserted plasmids. We identified multiple TFs whose regulatory changes increased thermotolerance, two TFs that eliminated pseudohyphal growth, and several TFs that increased betanin production. Analogous libraries can be designed to target any chosen group of genes and even all the genes. The libraries can be shared and reused, accelerating applied strain development projects and fundamental functional genomics research (TUNE^YALI^-TF kit and TUNE^YALI^-TF library are available via AddGene under catalog numbers #1000000255 and #217744).

## Significance

Three decades after metabolic engineering was established as a discipline, we are still very limited in our capabilities to design high-performing strains for industrial biotechnology rationally. Therefore, strain development projects require the testing of many engineering targets. To save time and costs, strain construction and testing could be executed in a high-throughput manner. Here, we present a methodology for high-throughput promoter replacement that allows tuning the expression of a selected group of genes in industrially important yeast *Yarrowia lipolytica*. The method will accelerate the strain development for industrial biotechnology and facilitate functional genomics research.

Metabolic engineering can deliver designer organisms for sustainable and cost-effective production of chemicals and recombinant proteins. Despite advances in predicting metabolic engineering targets through biochemistry, modeling, and omics data analysis (1), constructing high-performing strains still requires testing multiple hypotheses and iterative design-build-test cycles, making strain development costly and time-consuming. While biofoundries offer automated solutions for parallel strain construction and screening (2), they require significant investment and expertise. An alternative approach involves constructing diverse strain libraries using high-throughput (HTP) genome-editing methods and screening for improved performers. Various HTP genome editing techniques, based on recombineering, CRISPR/Cas, or sRNA/RNAi, have been demonstrated in *Escherichia coli* and *Saccharomyces cerevisiae* (3, 4). For example, Na et al. developed a synthetic small regulatory RNA (sRNA) library for *E. coli*, enabling high-throughput modulation of gene expression at the posttranscriptional level. This approach allowed the generation of large regulatory variant libraries and facilitated pathway optimization for enhanced production (5). Wang et al. introduced a multiplex genome engineering approach, MAGE, which enabled the rapid and scalable introduction of targeted mutations across multiple loci in *E. coli*. By generating large mutant libraries, this strategy accelerated the evolutionary optimization of metabolic pathways, significantly improving strain performance (6). Garst et al. integrated CRISPR-Cas9 with a multiplex genome-editing approach (CREATE), enabling precise and high-throughput mutation of multiple genomic loci. This method combines automated design of modular guide RNAs with barcode-enabled tracking, allowing for efficient, parallel editing of thousands of loci in the *E. coli* genome (7). These methods typically rely on oligonucleotide library synthesis but otherwise can be executed using standard molecular biology equipment, eliminating the need for extensive automation. While in bacteria, gene expression can be changed by modulating ribosome binding sites or short promoters, in yeast, gene expression tuning is more complex due to its significantly longer promoters. The median promoter length in *S. cerevisiae* is 455 bp, which complicates high-throughput gene expression modulation (8). Several strategies have been developed to address this challenge. Alper et al. constructed a synthetic promoter library using error-prone PCR, allowing systematic variation of gene expression levels by replacing the promoters of selected target genes (9). Global transcription machinery engineering (gTME) strategy has been applied to generate large variant libraries of global transcription regulators using error-prone PCR, improving ethanol tolerance and production in *S. cerevisiae* (10). Bowman et al. developed a CRISPR-dCas9-based library that targets promoter regions of 969 *S. cerevisiae* metabolic genes, enabling both up- and down-regulation of gene expression. This method facilitates expression modulation, aiding in the identification of optimal expression levels and gene targets for enhanced growth and production phenotypes (11). Wang et al. developed a CRISPR-Cas9 library that enables individual knock-outs of all 361 nonessential transporters in *S. cerevisiae* and applied this library to improve the production of *cis,cis*-muconic acid in a high-throughput manner, using fluorescent biosensor (12).

The genome editing of other yeasts is less well-developed. In particular, *Yarrowia lipolytica* has been gaining popularity in the last two decades as an industrial biotechnology cell factory. This Crabtree-negative yeast is used for the manufacturing of lipids, omega-3 fatty acids, steviol glycosides, pheromones, and other biochemicals (13). Various CRISPR-Cas9-based methods for targeted gene overexpression and deletion in *Y. lipolytica* are available (14, 15), but these methods are limited to a few targets. Of the high-throughput methods, whole-genome deletion libraries have been developed and applied to identify the targets for increased lipid accumulation and to improve the growth on various carbon sources (16, 17) (*SI Appendix*, Table S1). Also, a plasmid library expressing genomic DNA fragments was made and applied to identify genes improving propionate tolerance (18). This library does not edit the chromosomal genome and, therefore, can not be used for iterative strain improvement (18). We recently reported a method for combinatorial gene expression EXPRESS^YALI^, where up to three combinatorially assembled gene expression cassettes can be integrated into each yeast clone per round (19). The method, though, does not allow changing the expression of genes that are already present in the genome.

In this study, we present a targeted high-throughput strategy for gene expression tuning in *Y. lipolytica*. Our method utilizes a CRISPR-based promoter-swapping strategy to modulate gene expression, enabling precise control over gene expression levels while maintaining genetic stability. We constructed a library targeting 56 TFs, adjusting their expression to seven different levels. This strategy can provide insights into relationships between transcription factors (TFs) and desired phenotypes, offering a scalable and efficient solution for fine-tuning metabolic pathways in *Y. lipolytica*. By expanding the genetic toolbox for this yeast, our approach enhances its potential as a robust host for industrial applications.

## Results

### Development of a Method for Scarless Promoter Replacement.

**Fig. 1.**
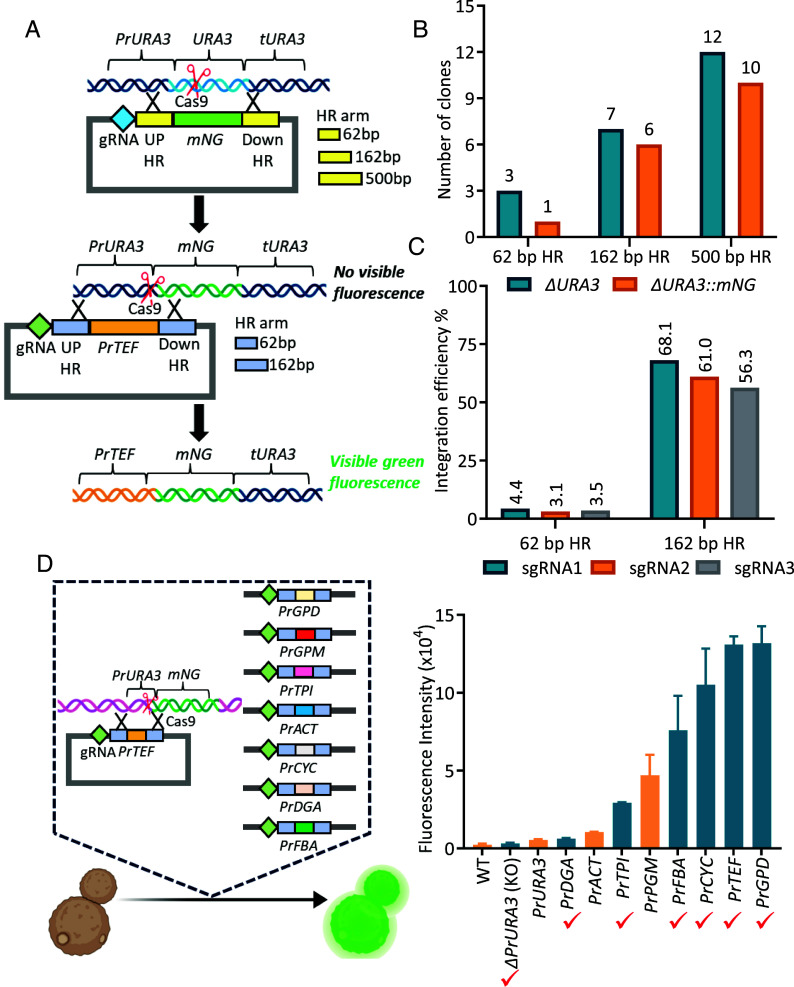
Development of the method for effective promoter replacement in *Yarrowia lipolytica*. (*A*) First, the *URA3* gene encoding orotidine 5-phosphate decarboxylase was replaced with gene *mNG* encoding fluorescent protein mediated by CRISPR/cas9 restriction. Homologous arms of different lengths were used. Then, the *URA3* promoter upstream of the *mNG* gene was replaced with a stronger *TEF* promoter. (*B*) Efficiency of *URA3* deletion and replacement of *URA3* gene with *mNG* gene. (*C*) Efficiency of replacing *PrURA3* promoter with *PrTEF*. (*D*). Design for replacing *PrURA3* with various alternative promoters to regulate *mNG* expression. The fluorescence intensity of *mNG* driven by each promoter was measured via flow cytometry. Promoters marked with the symbol ✓ were selected to construct the TUNE^YALI^-TF library.

In library-scale genome editing, ensuring the correct pairing of the sgRNA and its corresponding repair element is essential for achieving efficient editing. Traditional CRISPR-Cas9 methods in *Y. lipolytica* typically rely on cotransforming linear DNA repair elements with an sgRNA plasmid (14, 15, 20). However, this approach is impractical for library-size genome editing. When a pool of linear repair elements is combined with a pool of sgRNA vectors, the probability of matching elements entering the same cell is low, leading to reduced editing efficiency and unintended repair through nonhomologous end-joining. To overcome these challenges, we developed a system in which both the sgRNA and its corresponding repair template are encoded within a single plasmid, ensuring efficient editing and the correct pairing of the sgRNA with its corresponding repair element. Therefore, we first evaluated the efficiency of genome editing in *Y. lipolytica* when sgRNA and repair templates were provided on a single plasmid. We used a sgRNA targeting the *URA3* locus and a repair template that contained up- and downstream homologous recombination (HR) arms, along with a coding sequence (CDS) for the green fluorescent protein mNeonGreen (*mNG*) (Fig. 1*A*). The HR arms of three lengths were tested: 62, 162, and 500 bp (Fig. 1*A*). As expected, the genome editing efficiency increased with longer HR elements (Fig. 1*B*). Several clones with disruption of the *URA3* gene were not fluorescent, indicating that repair occurred via nonhomologous end joining instead of homologous recombination with *mNG* cassette. While the highest efficiency was achieved with 500 bp overhangs, using such long sequences would significantly increase synthetic DNA costs. Therefore, we chose to continue with 62 and 162 bp overhangs.

Next, we designed an approach for constructing plasmids that carry promoters between up- and downstream HR elements, thus enabling promoter replacement of the target gene. The proposed workflow is as follows: Synthetic DNA consisting of a target-specific sgRNA and target-specific up-and-down HR elements are designed. The sgRNA is designed to target the promoter region of the target gene. The upstream HR element matches the region upstream of the native promoter of the target gene. The downstream HR element matches the start of the CDS of the target gene. A double SapI restriction site is added in between the HR elements, allowing for the insertion of a promoter element. The 3-bp overhang generated by SapI, corresponding to a start codon (ATG), prevents the formation of scars between the promoter and the downstream homologous recombination (HR) element, which is a critical CDS boundary. 20 bp Gibson assembly homology arms are added to each side of the synthetic construct. The length of the whole synthetic element is 300 bp (for 62 bp HR) or 500 bp (for 162 bp HR). The designed DNA constructs are synthesized and cloned into a plasmid backbone individually via Gibson assembly. The resulting plasmids can be mixed individually or in groups with a desired number of promoters, and promoters can be inserted between the HR elements by Golden Gate using the SapI enzyme.

We designed sgRNA and HR elements targeting the *URA3* promoter to validate this approach and cloned the *TEF1* promoter between the HR elements (Fig. 1*A*). As sgRNA efficiencies may differ depending on the target site, we designed three different sgRNAs. We then transformed these plasmids into the strain with the *URA3* promoter-controlled fluorescent *mNG* expression (*ΔURA3::mNG* strain ST14141) (Fig. 1*A*). Clones where the *URA3* promoter was replaced with the *TEF1* promoter had significantly higher fluorescence, which allowed us to evaluate the genome editing efficiency (*SI Appendix*, Fig. S1). To compare the integration efficiency of homologous arms with lengths of 62 bp and 162 bp, we quantified both the total number of transformants and the number of transformants exhibiting green fluorescence, indicating successful modification. The use of 162 bp homologous arms resulted in a significantly higher number of total colonies—reaching the hundreds—as well as a greater number of fluorescent colonies. In contrast, the 62 bp homologous arms yielded substantially fewer fluorescent colonies, confirming that the longer homologous arms enhance homologous recombination efficiency. The editing efficiency for 162 bp HR elements ranged from 56.3 to 68.1%, whereas the efficiency for 62 bp HR elements was markedly lower, with only 3.1 to 4.4% of clones exhibiting promoter replacement (Fig. 1*C*). Using 62 bp elements would allow the cheaper synthesis of 300 bp-long sgRNA-repair constructs on DNA arrays. However, the trade-off would be a low percentage of edited clones. We, therefore, chose to continue the work with 162 bp HR arms, resulting in 500 bp-long sgRNA-repair constructs that can be cost-effectively synthesized as gene blocks.

To enable precise regulation of gene expression, we characterized eight native *Y. lipolytica* promoters by cloning them upstream of fluorescent protein *mNG* in *ΔURA3::mNG* strain (ST14141) and measuring the fluorescence of the resulting yeast strains by flow cytometry (Fig. 1*D*). We selected six promoters of varying strength for use in the toolbox (*PrDGA*, *PrTPI*, *PrFBA*, *PrCYC*, *PrTEF*, *PrGPD*).

### Construction of the High-Throughput *Y. lipolytica* Genome Editing Toolkit TUNE^YALI^.

To validate the promoter replacement (TUNE^YALI^) approach, we decided to modulate the expression of 60 TFs. The rationale was that such a library (TUNE^YALI^-TF library) could be applied for engineering various phenotypes rather than a library targeting a specific metabolic pathway.

First, we compiled a list of 270 TFs based on genome annotation and literature (21–27) (Dataset S1). Next, we extracted transcriptome and proteome data for these TFs from two studies (28, 29) and selected 60 TFs that are either highly expressed (at gene or protein levels) in *Y. lipolytica* or identified in the literature as critical regulators of essential cellular processes and pathways (Dataset S1). Subsequently, we designed 500 bp sgRNA-repair elements targeting the promoter regions of these TFs as described above (*SI Appendix*, Fig. S2) and cloned them to create 56 basic plasmids, each capable of removing the promoter upstream of the targeted TF (*SI Appendix*, Fig. S3). The cloning of 4 elements failed. Then, we mixed all 56 plasmids and incorporated six selected promoters as selected in the previous section in a single-pot Golden Gate reaction (*SI Appendix*, Fig. S3). The targeted library diversity of 56 × 7 = 392 constructs was confirmed by Nanopore sequencing (*SI Appendix*, Fig. S4 and Dataset S2). The analysis showed an average coverage depth of 222.99X, ensuring that each position in the library was covered multiple times for reliable results. The coverage rate of 99.9914% indicates that nearly the entire library was successfully sequenced. Furthermore, the abundance of each plasmid was assessed, revealing minimal variation across plasmids. Detailed coverage and abundance data for each plasmid can be found in Dataset S2.

### Application of the TUNE^YALI^ Toolkit for Morphological Engineering of *Y. lipolytica*.

**Fig. 2.**
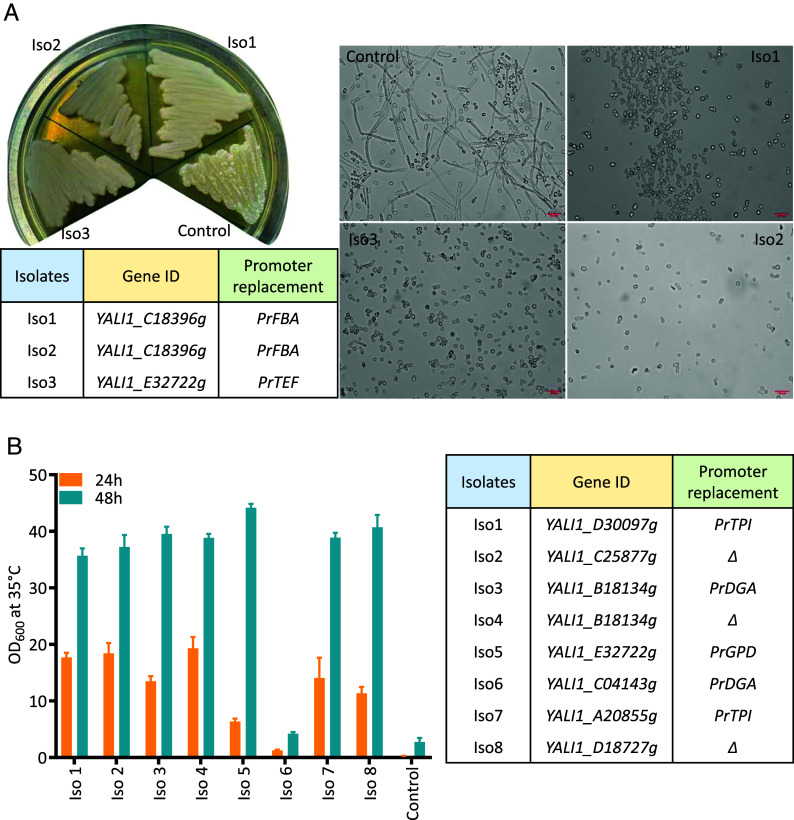
Engineering morphology and thermotolerance by modulation of transcription factor (TF) expression using TUNE^YALI^-TF library. (*A*) Microscopy images of selected *Y. lipolytica* isolates with altered morphologies and their genotypes. (*B*) Growth of *Y. lipolytica* isolates with enhanced temperature tolerance at 35 °C and their genotypes. *Δ* indicates that the native promoter of the target genes was deleted instead of being replaced with an alternative promoter.

In fermentation processes, pseudohyphae formation is often observed in *Y. lipolytica*, leading to increased foaming and a reduced oxygen transfer rate (30, 31). The TF *MHY1*, part of the cAMP protein kinase A (PKA) pathway, has been identified as a critical regulator of the morphological transition from yeast to mycelium form (25, 32, 33). However, the roles of many other TFs remain largely unexplored. To investigate this further, we utilized our TUNE^YALI^-TF toolkit for morphological engineering in *Y. lipolytica* to optimize the strain and identify morphology-related TFs. We transformed the parental *Y. lipolytica* strain ST6512, derived from W29 (ATCC 20460) and harboring Cas9 in the *KU70* locus (34), with the TUNE^YALI^-TF library and recovered the clones in YPD medium for 2 h. Then, we plated the transformants on YPD agar plates supplied with hygromycin for selection. After two days, we observed three colonies with a smooth mucoid surface among over a thousand colonies (Fig. 2*A*). Microscopic examination confirmed a morphological change, showing a complete absence of pseudohyphae (Fig. 2*A*). When these colonies were inoculated into 1 mL of YPD liquid medium and cultured overnight, the medium turned dark (*SI Appendix*, Fig. S5). *Y. lipolytica* is known to produce black pyomelanin pigments under stress conditions such as oxidative stress, pH changes, or specific environmental factors, suggesting that pyomelanin production likely caused the dark coloration in these colonies. The modified TFs were identified by PCR-amplification of the variable fragment from the transformed plasmid and Sanger sequencing. Of the three isolates, two had identical mutations, with the native promoter of *YALI1_E32722g* (TF23) replaced by *PrTEF*, while one colony had the promoter of *YALI1_C18396g* (TF48) replaced by *PrFBA*. Neither of the two TFs has been previously associated with morphology. *YALI1_C18396g* (TF48) is downregulated under alkaline pH (35), whereas *YALI1_E32722g* (TF23) is similar to *S. cerevisiae GCN4* transcriptional activator of amino acid biosynthetic genes (29) (Table 1). Further research is required to elucidate the relationship between these two TFs and yeast morphology, as well as their roles in pyomelanin production.

**Table 1.** Transcription factors (TFs) modulated in this study (TUNE^YALI^-TF library)

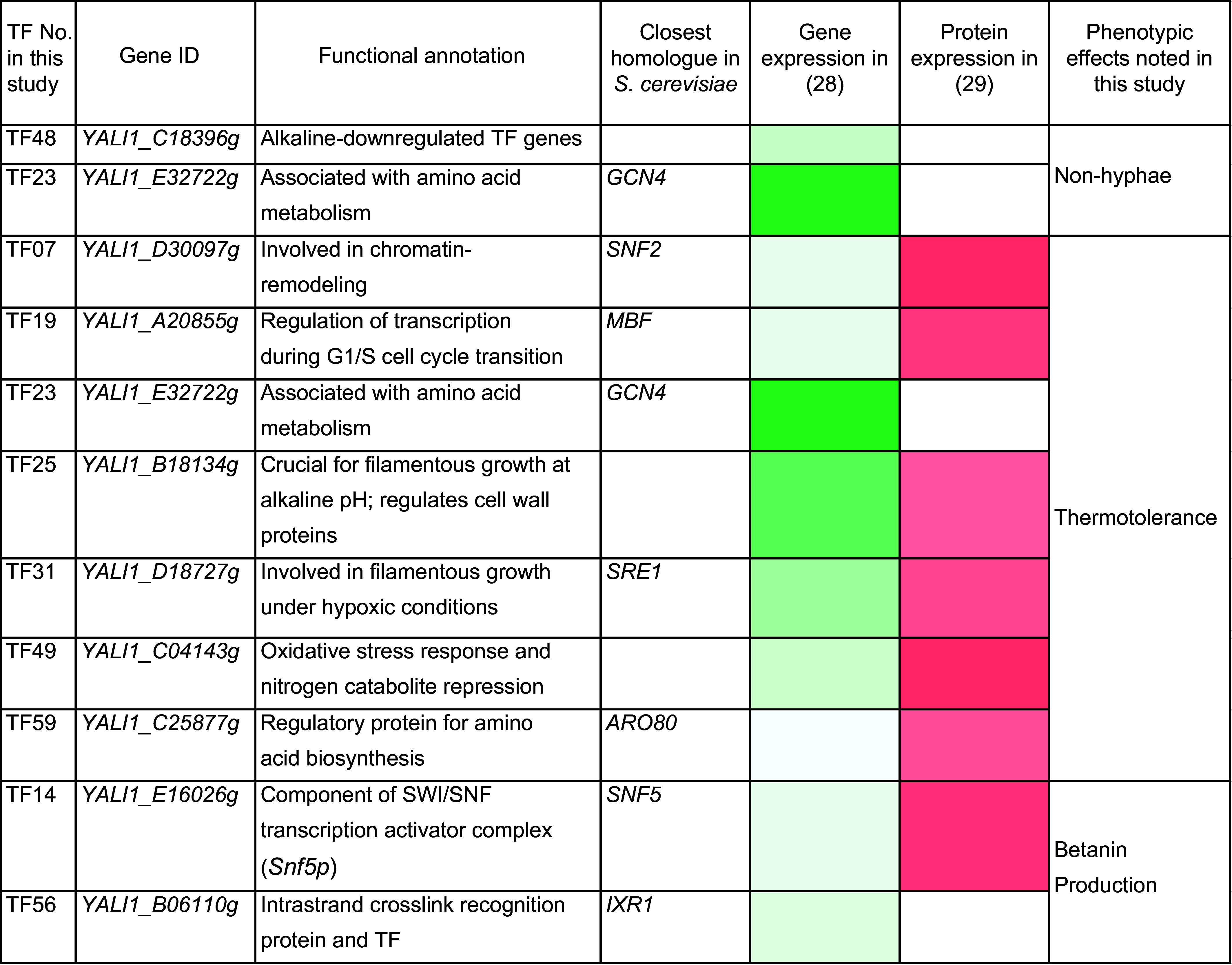

The intensity of the color represents the level of expression.

### Application of the TUNE^YALI^ Toolkit for Tolerance Engineering of *Y. lipolytica*.

We further investigated the application of the TUNE^YALI^ toolkit for tolerance engineering. Yeast strains can be sensitive to stressors commonly occurring in large-scale fermentations, such as increased temperature, pH fluctuations, limited oxygen, and others (36, 37). Stress-regulated TFs play a key role in managing these responses (38). In this study, we investigated whether modulating any TFs from our library can improve the thermotolerance of *Y. lipolytica*. To achieve this, we introduced the TUNE^YALI^-TF library into the same parental strain ST6512 as above. Transformants were plated on YPD plates with hygromycin B and incubated at 33 °C. After two days, emerging colonies were transferred to tubes with 1 mL of liquid YPD and cultivated with shaking at 35 °C. Ultimately, we obtained eight isolates capable of growing at 35 °C (Fig. 2*B*). Interestingly, we found that each strain had distinct modifications involving different TFs. These included the replacement of the native promoter of *YALI1_D30097g* (TF07) with *PrTPI*, *YALI1_B18134g* (TF25) with *PrDGA*, *YALI1_E32722g* (TF23) with *PrGPD*, *YALI1_C04143g* (TF49) with *PrDGA*, and *YALI1_A20855g* (TF19) with *PrTPI*. Additionally, some strains had deletions of the native promoters of *YALI1_D18727g* (TF31), *YALI1_B18134g* (TF25), or *YALI1_C25877g* (TF59) (Fig. 2*B*). These TF genes were associated with processes such as amino acid biosynthesis, oxidative stress response, nitrogen catabolite repression, and filamentous growth, but none were linked to thermotolerance (Table 1 and Dataset S1). Notably, the gene *YALI1_D30097g*, modulated in one of the thermotolerant *Y. lipolytica* isolates, is similar to *S. cerevisiae*’s transcription regulatory protein Snf2p as a key component of the SWI/SNF complex, which plays an essential role for thermotolerance development of *S. cerevisiae* (39). We have measured the growth time course of *Y. lipolytica* thermotolerant isolates and the control strain (W29) at 35 °C in microtiter plates. All the isolates had higher maximum specific growth rates (up to 55% higher) and reached higher optical density after 20 h than the control strain (*SI Appendix*, Fig. S6).

### Application of the TUNE^YALI^ Toolkit for Enhanced Betanin Production in *Y. lipolytica*.

**Fig. 3.**
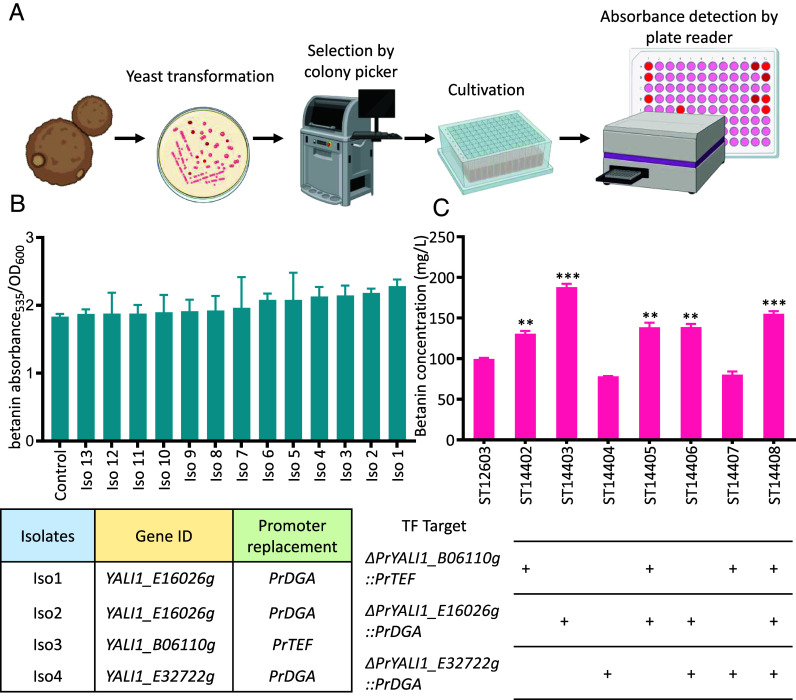
Application of the TUNE^YALI^-TF library for betanin production. (*A*) Workflow for screening *Y. lipolytica* strains for improved betanin production. (*B*) Specific betanin production in selected isolates with enhanced betanin production. Genotypes of the top 4 isolates. (*C*) HPLC-measured betanin titer in reverse-engineered strains. Statistical significance was analyzed using a two-tailed Student’s *t* test (*P < 0.05, **P < 0.01, ***P < 0.001), with comparisons to the control strain ST12603.

The toolkit TUNE^YALI^-TF could also be useful for engineering cell factories to increase the production of various metabolites. We chose to apply it to optimize the production of the red pigment betanin for the ease of screening. Betanin is a red-violet pigment present in some Caryophyllales plants and higher fungi (40, 41), it is commonly used as a food colorant. We introduced the TUNE^YALI^-TF library into *Y. lipolytica* strain ST12603, which was previously rationally engineered for betanin production (42). The transformation mix was plated on antibiotic selection plates, and 128 of the reddest colonies, selected from over a thousand colonies, were transferred into liquid medium in 96-deep-well plates by a colony-picking robot (Fig. 3*A*). After 72-h cultivation, we measured optical density (OD_600_) and betanin absorbance using a microtiter plate reader and then calculated the normalized betanin absorbance [nAb(_betanin_) = betanin absorbance_535_/OD_600_] (Dataset S3). The increase of nAb(_betanin_) was in the range of 2.19 to 24.51% (Fig. 3*B*). Using PCR and Sanger sequencing as above, we identified the targeted TFs for the top four clones. The top four clones had a change in *YALI1_E16026g, YALI1_B06110g,* or *YALI1_E32722g*, where their native promoters were replaced by *PrDGA*, *PrTEF,* and *PrDGA*, respectively. The same *PrDGA−YALI1_E16026g* genotype occurred in two clones. *YALI1_E16026g* (TF14) shares similarity with *S. cerevisiae*’s Snf5p, a subunit of the SWI/SNF chromatin-remodeling complex that is recruited by activators Hap4p and Gcn4p (43) (Table 1). The closest homolog of the *YALI1_B06110g*-encoded protein (TF56) in *S. cerevisiae* is Ixr1p, a transcriptional repressor involved in the hypoxic response (44) (Table 1). The third target, *YALI1_E32722g* (TF23), encodes a protein similar to *S. cerevisiae* Gcn4p, a global regulator of general amino acid control (45) (Table 1). All these three TF targets that improved betanin production are nonobvious and could not be found rationally. Further studies are required to elucidate how they influence betanin biosynthesis.

We performed reverse engineering to confirm the roles of identified TFs in enhancing betanin production and also tested the effects of combining these mutations. We constructed seven new strains (ST14402 to ST14408) with individual promoter replacements, including **∆*PrYALI1_B06110g* (TF56)*::PrTEF*, **∆*PrYALI1_E16026g* (TF14)*::PrDGA*, **∆*PrYALI1_E32722g* (TF23)*::PrDGA*, as well as combinations of these modifications, using ST12603 as the parental strain, which currently holds the highest reported betanin titer (42). These strains, alongside the control strain ST12603, were cultured in a minimal medium, and their growth time course was measured in microtiter plates (*SI Appendix*, Fig. S7). Compared to the control strain, all engineered strains exhibited a lower maximum specific growth rate. Additionally, double and triple gene modifications tended to extend the lag phase, with ST14406 (14 h lag) and ST14408 showing the most pronounced delays. These results suggest a trade-off between betanin production and growth. Following 48 h of cultivation in 2 mL minimal medium using 24 deep-well plates, betanin titer was determined using HPLC. Modifications **∆*PrYALI1_B06110g* (TF56)*::PrTEF* and **∆*Pr YALI1_E16026g* (TF14)*::PrDGA* significantly increased betanin titer to 130 mg/L and 188 mg/L, respectively, surpassing the control’s 99 mg/L (ST12603) (Fig. 3*C*). However, the modification *∆PrYALI1_E32722g* (TF23)*::PrDGA* resulted in a reduction in betanin titer (78 mg/L). In addition, the combination of these mutations did not yield a synergistic effect.

From an industrial biotechnology perspective, product titer is the primary determinant of COGS, provided that rate and yield remain sufficient. Techno-economic analysis (TEA) and life-cycle assessment (LCA) of the control strain ST12603 (42) indicate that increasing betanin titer directly enhances process economics by reducing volumetric production costs. Our findings demonstrate that TF tuning can effectively improve betanin production in *Y. lipolytica*, reinforcing its potential as a viable host for industrial applications where strain optimization enhances production efficiency and economic feasibility.

## Discussion

Strain development programs in industrial biotechnology are costly and time-consuming, largely due to the limited ability to rationally engineer strains with desired phenotypes. Multiple metabolic engineering targets can be identified through metabolic modeling, omics data analyses, and biochemical considerations (46, 47). However, only a small fraction of these targets typically yield improvements in product titer, rate, or yield. Even then, the improvements are often marginal. Moreover, combining successful targets introduces the risk of antagonistic interactions, turning strain engineering into a complex and challenging combinatorial problem.

These challenges can be addressed by adopting high-throughput strain engineering methodologies. Instead of parallel construction of individual strains, strain libraries can be constructed in a single batch and then screened to identify the best performers. In this way, hundreds of hypotheses can be tested simultaneously. The best performers are then subjected to the next rounds of library-size engineering and selection until the desired strain performance is achieved. If higher-throughput screening methods are available, the library size can be scaled accordingly. For instance, fluorescence-activated cell sorting (FACS) enables rapid screening when the product itself is fluorescent (48) or when a fluorescent signal is generated via a biosensor (49–51). Additionally, if the desired phenotype is linked to growth—such as tolerance to an inhibitor or production-coupled growth—larger libraries can be screened using growth-based selection strategies (52, 53). Some rapid mass spectrometry (MS)-based methods are emerging as viable options for high-throughput screening (54, 55). If high-throughput screening methods are not feasible, the strain library size could be adjusted to match the processing capacity of the available methods or the library can be randomly sampled. The target sets can be designed to integrate heterologous genes (56), to create mutations of a specific flux-controlling enzyme (57, 58), or to change the gene expression.

In this study, we developed a methodology for high-throughput engineering of industrially important oleaginous yeast *Y. lipolytica* (TUNE^YALI^). A similar principle can be applied to other organisms where genome integration via homologous recombination can be accomplished. To illustrate the method, we created a TUNE^YALI^-TF set targeting 56 TFs and applied it to enhance betanin production and improve thermotolerance and morphology phenotypes. Modulating TF expression resulted in diverse phenotypic outcomes, highlighting the complexity of regulatory networks in *Y. lipolytica*. *YALI1_E16026g*, which improved betanin production, is a homolog of *S. cerevisiae SNF5*, a subunit of the SWI/SNF chromatin remodeling complex. Deletion of the *SNF5* gene in *S. cerevisiae* impairs growth (59, 60). Possibly, the change of the native to a strong *DGA* promoter increased the expression of *SNF5* and, consequently, the transcription of the genes involved in betanin biosynthesis. Another TF that improved betanin production was *YALI1_B06110g*, homologous to *S. cerevisiae IXR1*, which is involved in adaptation to hypoxia and oxidative stress (44). Betanin biosynthesis involves an oxygen monooxygenase P450 enzyme, which makes it highly sensitive to oxygen and heme availability and may be influenced by *IXR1*. A transposon mutagenesis screen of *Y. lipolytica* previously identified mutations of seven genes that resulted in defective hyphal formation (61), of which one was a TF *SNF5*, also present in our library. Interestingly, *SNF5* did not come up among the morphology mutants in our library, but two other TFs gave the desired nonhyphal morphology. Neither of these two TFs (*YALI1_C18396g* and *YALI1_E32722g*, *GCN4* homolog) were previously associated with morphological changes. Of the seven TFs whose modulation resulted in thermotolerance, two, *SNF2* and *GCN4*, are associated with thermotolerance in *S. cerevisiae*. *SNF2* deletion mutant showed better survival under 50 °C heat shock (62) than the wild type. Our *Y. lipolytica* mutant Iso1 had *SNF2* promoter swap to *TPI* promoter, which was one of the weaker promoters in our screen and could potentially result in *SNF2* downregulation. Reduced *GCN4* activity mutant of *S. cerevisiae* was less resistant to 48 °C heat stress than wild type if subjected to 37 °C adaptation (63). Our Iso 5 thermotolerant mutant had *GCN4* promoter swapped with *GPD* promoter, the strongest in our promoter set, which may have resulted in its overexpression. These hypotheses could be further investigated by transcriptomics. Notably, the mechanisms by which TF expression modulation changed the cellular phenotypes remain well understood, highlighting the power of this method to identify nonobvious metabolic engineering targets.

The TUNE^YALI^ system provides a flexible and scalable approach for modulating gene expression in *Y. lipolytica*. Targeting specific gene subsets enables precise control over cellular functions to optimize strain performance. In this study, we applied the TUNE^YALI^-TF library to regulate 56 TFs, but the approach can be adapted to different gene sets depending on project goals.

One of the key advantages of the TUNE^YALI^ system is its potential for iterative application. To explore this, we reintroduced the TUNE^YALI^-TF library into the top betanin-producing strain identified in our initial screening. However, no further improvement was observed (Dataset S4), suggesting that transcriptional regulation was no longer the primary flux-controlling element at this stage. Instead, constraints such as precursor availability or betanin biosynthetic enzyme activity may play a more significant role in enhancing production. To address these limitations, future efforts should focus on constructing new TUNE^YALI^ libraries targeting alternative gene sets to further optimize strain performance. Beyond betanin production, the TUNE^YALI^-TF library has demonstrated its potential to enhance multiple industrially relevant phenotypes. We have shown its applicability in improving thermotolerance and morphology. These findings highlight the broad utility of TUNE^YALI^ libraries as a powerful tool for strain engineering across diverse applications.

The main limitation of the TUNE^YALI^ system is that only a single genome edit is introduced per clone per transformation. To obtain combinations of targets, the library would need to be transformed over several rounds. Another limitation is that the method only performs promoter removal or replacement, so if it is desired to introduce additional genes into the genome, other methods should be used, for example, EXPRESS^YALI^ which allows the combinatorial introduction of gene expression cassettes (19). Looking ahead, we envision the expansion of the TUNE^YALI^ toolkit to other *Y. lipolytica* genes, potentially even all the genes, facilitating the strain development in industrial biotechnology and advancing the functional genomics of *Y. lipolytica*.

## Materials and Methods

### Plasmid Construction and *E. coli* Transformation.

sgRNA sequences for substituting *URA3* with the green fluorescent protein *mNG* and native promoters of *URA3* with different promoters in *Y. lipolytica* were designed using the CHOPCHOP (64) website. Homologous arms of varying lengths (62 bp, 160 bp, and 500 bp) for these substitutions were amplified from the *Y. lipolytica* genome using Phusion Hot Start II High-Fidelity PCR Master Mix (Thermo Scientific™, F565S). The plasmids were constructed by assembling these bioparts through the Gibson assembly method. DNA strings of promoters were synthesized by Twist Bioscience (USA) and cloned into the PCR-Blunt II TOPO vector (Thermo Scientific™, 451245). Different promoters were inserted between the upstream and downstream homologous arms by the SapI Golden Gate assembly following the methodology outlined in Shaw’s study (65). Each component was standardized to an equimolar concentration of 50 fmol/mL (50 nM) before assembly. The Golden Gate assembly was performed using the following components: 0.5 μL of the backbone vector, 1.5 μL of each insert plasmid, 1 μL of T4 DNA ligase buffer (NEB, B0202S), 1 μL of T7 DNA ligase (NEB, M0318S), 1 ul of SapI (NEB, R0569S), and water to a total volume of 20 μL. These reaction mixtures were subjected to a thermocycling program set as 25 cycles of 37 °C for 2 min and 16 °C for 5 min, followed by a digestion step at 60 °C for 10 min and a final heat inactivation step at 80 °C for 10 min. The entire Golden Gate reaction mixture was transferred into *E. coli* strain DH5α. Transformed *E. coli* cells were selected on LB agar plates containing kanamycin (50 µg/mL) or ampicillin (100 μg/ mL). All the plasmids used in this study are summarized in *SI Appendix*, Table S3.

### Toolkits Construction.

sgRNA-PAM sequence (23 bp) for 60 TFs of the background yeast ST6512, a W29 (ATCC 20460) strain harboring Cas9 in the *KU70* locus (34), was designed using CHOPCHOP through typing in the gene IDs of the chosen targets and selecting promoter as the target specific region of the gene. We prioritized sgRNAs with minimal mismatches, an efficiency score greater than 60%, and a position within −200 bp of the start codon of targeted genes. The selected sgRNA sequences are detailed in Dataset S1. We designed upstream and downstream homologous arms, each 162 bp long, spanning from −500 bp and 0 bp relative to the gene’s start codon. These arms, generated via a custom Python script, exclude the SapI restriction site. DNA fragments (500 bp) consisting of the sgRNA-scaffold, homologous arms, SapI site for promoter insertion, and overhangs at both ends, were synthesized by Twist Bioscience (USA). The library plasmid’s backbone was amplified using Phusion Hot Start II High-Fidelity PCR Master Mix (Thermo Scientific™, F565S) and assembled with the 500 bp DNA fragments using the Gibson Assembly® Master Mix (New England BioLabs, E2611L). The assembled construct was then introduced into *E. coli* strain DH5α and cultivated on LB agar plates with suitable antibiotics. Plasmid isolation followed the protocol of the mini prep kit (MACHEREY-NAGEL GmbH, Germany) and was verified by Sanger sequencing (Eurofins Scientific SE).

The verified *E. coli* transformants were individually cultured overnight in 2 mL of selective LB medium. The cultures were then pooled for plasmid isolation and combined with various promoters using the SapI Golden Gate reaction, as described in Section 2.2. The reaction underwent 120 cycles at 37 °C for 2 min and 16 °C for 5 min. The entire mixture was then introduced into *E. coli* strain DH5α and incubated overnight on large square plates (Avantor®, ANIC05.40.18PAI) containing LB agar with ampicillin. The plates were rinsed 3 to 4 times with 2 to 3 mL of sterilized water, and each rinse was transferred to a 50 mL Falcon tube to collect the bacteria. Finally, the colonies collected from these washes were pooled together. We mixed each 100 μL aliquot with an equal volume of 50% sterilized glycerol for storage at -80 °C. To retrieve aliquots from the library, we revived the stored *E. coli* on square LB selective plates. Plasmids libraries were then collected using the washing method and isolated with the miniprep kit.

### Nanopore Sequencing and Data Processing.

The diversity of the library and the coverage of the design space were determined by Nanopore sequencing. DNA libraries for Nanopore sequencing were prepared according to the manufacturer’s instructions, tagging with unique barcodes using the SQK-RBK114.96 kit. These libraries were loaded into an R10.4.1 flow cell for sequencing. The GridION Mk1 device captured raw sequencing data in FAST5 format.

Nanopore sequencing raw data were processed into FASTQ format sequence reads using the MinKNOW (15.3.0) onboard Dorado (7.1.4 + d7df870c0) basecaller with the Super accuracy model (Oxford Nanopore Technology). These reads were then mapped to the constructed reference plasmid library using the Minimap2 aligner tool (available at https://github.com/lh3/minimap2). Subsequently, Samtools (accessible at https://github.com/samtools/samtools) was utilized to calculate the average coverage, coverage rate, and abundance for each plasmid.

### Yeast Construction and Culture Conditions.

This study utilized strains originating from ST6512, a derivative of W29 (NRRL Y-63746) containing Cas9 at the *KU70* locus (34). All the strains were constructed using the CRISPR-Cas9 genome editing method. Yeast transformation was carried out using a standard lithium acetate technique (66). Yeast transformants were selected on either YPD (10 g/L yeast extract, 20 g/L peptone, 20 g/L glucose) with 400 mg/L hygromycin B (Invitrogen, 10687010) or YNB minimal medium (6.8 g/L yeast nitrogen base without amino acids, 50 mM phosphate buffer pH 6.8, 20 g/L glucose) with/without 400 mg/L hygromycin B or YNB +Ura -Leu (YNB minimal medium with 76 mg/L uracil). Successful incorporation of genes was verified through yeast colony PCR using the Phire Plant Direct PCR Master Mix (F160S, Thermo Fisher). All the strains used in this study are listed in *SI Appendix*, Table S4.

For betanin production, *Y. lipolytica* strains were cultivated in a mineral medium (67) with pH 6, containing the following components per liter: 7.5 g (NH_4_)_2_SO_4_, 14.4 g KH_2_PO_4_, 0.5 g MgSO_4_ 7H_2_O, 22 g dextrose, 2 mL trace metals solution (3.0 g/L FeSO_4_·7H_2_O, 4.5 g/L ZnSO_4_·7H2O, 4.5 g/L CaCl_2_·2H_2_O, 0.84 g/L MnCl_2_·2H_2_O, 0.3 g/L CoCl_2_·6H_2_O, 0.3 g/L CuSO_4_·5H_2_O, 0.4 g/L Na_2_MoO_4_·2H_2_O, 1.0 g/L H_3_BO_3_, 0.1 g/L KI, and 19.0 g/L Na_2_EDTA·2H_2_O), and 1 mL vitamins (0.05 g/L D-biotin, 0.2 g/L p-aminobenzoic acid, 1.0 g/L calcium D-pantothenate, 1.0 g/L thiamin–HCl, 1.0 g/L pyridoxin–HCl, 1.0 g/L nicotinic acid, and 25.0 g/L myo-inositol).

To screen for high thermotolerance strains, the TUNE^YALI^ plasmid library was transferred into the background strain ST6512 following a standard lithium acetate technique, then plated on YPD plates with hygromycin B (400 mg/L) at 33 °C. After two days, yeast colonies emerged and were transferred to 1 mL of YPD and cultivated at 35 °C to test growth.

### Promoter Intensity Detection.

Fresh yeast colonies were first grown overnight in 1 mL YNB minimal medium at 30 °C, 250 rpm to evaluate promoter strength. These cultures were then inoculated into 96-deep-well plates containing 500 μL YNB minimal medium, ensuring an initial OD of 0.2. After overnight incubation, 200 μL of the culture was used for fluorescence measurements by flow cytometry (NovoCyte Quanteon, Agilent, USA). The excitation and emission wavelengths for the green fluorescent protein *mNG* were 485 nm and 535 nm, respectively.

### Automatic Colony Picker.

The TUNE^YALI^ library was introduced into the betanin-producing strain ST12603 (42). After two days of incubation at 30 °C, yeast colonies were observed on YPD agar plates supplemented with hygromycin B. These colonies were then transferred to Nunc™ OmniTray YPD agar plates (Thermo Scientific™) using a PIXL robot (Singer Instruments) and picked automatically through the PIXL imaging software (version 2.21.1001.4).

### Optical Density and Betanin Absorbance Measurements.

Colonies picked by the robot from YPD agar plates with hygromycin B were relocated to 96-deep-well plates filled with 500 μL YPD medium and hygromycin B. After overnight incubation, they were moved to new 96-deep-well plates containing YNB minimal medium with hygromycin B and cultured for an additional 96 h, setting the stage for betanin quantification. The optical density (OD_600_) and betanin absorbance (535 nm) measurements were conducted using a BioTek Synergy MX plate reader (Holm & Halby). A 20 μL aliquot of the yeast culture, diluted tenfold, was measured in 96-well clear-bottom plates (Corning), using the diluted medium as a blank for absorbance measurements.

### Target Verification in Screened Strains.

Episomal plasmids were isolated from the screened strains with the increased betanin production using the Zymoprep™ yeast plasmid miniprep kit (Zymoprep™, USA). The plasmids were introduced into *E. coli* DH5α for purification and subsequently analyzed through Sanger sequencing performed by Eurofins Genomics (Germany). In cases where target verification in yeast strains was unsuccessful, a yeast colony PCR approach was employed. This method amplified a fragment encompassing the 20 bp sgRNA, sgRNA scaffold, upstream and downstream homologous arms, and the inserted promoter using the Phire Plant Direct PCR Master Mix (F160S, Thermo Fisher Scientific). The resulting PCR products were then sequenced using Sanger sequencing (Eurofins Genomics, Germany) for confirmation.

### Betanin Extraction and Quantification by HPLC.

For betanin extraction and detection, as described in our previous work (56), 1 mL of culture was placed in a 2 mL microtube with glass beads and lysed using a cell disruptor (Percellys 24). After centrifugation at 10,000 g for 10 min at 4 °C, the supernatant was analyzed for betanin content using HPLC. The HPLC analysis, conducted on a Dionex UltiMate 3000 system with a Zorbax Eclipse Plus C18 column, started with a solvent mixture of 98% phase A (0.1% formic acid) and 2% phase B (acetonitrile), followed by a gradient reaching 98% phase B. The column temperature was maintained at 30 °C, with a 10 μL injection volume and 1 mL/min flow rate. Calibration curves for quantification were established with various betanin standards (Sigma-Aldrich, 901266).

### Microscopy of Yeast Cells.

Single clones were cultured on YPD agar plates to examine their colony morphology. The cells were washed with 1x PBS and observed using a LEICA DM 4000B microscope under a 40× objective lens.

## Supplementary Material

Appendix 01 (PDF)

Dataset S01 (XLSX)

Dataset S02 (XLSX)

Dataset S03 (XLSX)

Dataset S04 (XLSX)

## Data Availability

All study data are included in the article and/or supporting information.
