# Supplementary material for: High-throughput metabolic engineering of Yarrowia lipolytica through gene expression tuning
Source: Proc Natl Acad Sci U S A. 2025 Jun 3;122(23):e2426686122. doi: 10.1073/pnas.2426686122 (PMC12168020; doi:10.1073/pnas.2426686122)
Supplement: Supplementary file 1 — Appendix 01 (PDF) [file pnas.2426686122.sapp.pdf]

High-throughput metabolic engineering of *Yarrowia lipolytica* through gene expression tuning

Wei Jiang[1], Shengbao Wang[1], Daniel Ahlheit[1,2], Tommaso Fumagalli[1,3], Zhijie Yang[1], Shreemaya Ramanathan[1], Xinglin Jiang[1], Tilmann Weber[1], Jonathan Dahlin[1], Irina Borodina[1]*

[1]The Novo Nordisk Foundation Center for Biosustainability, Technical University of Denmark, DK-2800, Kgs. Lyngby, Denmark

[2]Department of Sustainable Biotechnology, Aalborg University, Fredrik Bajers Vej 1, DK-9220, Aalborg East, Denmark

[3]Department of Biotechnology and Biosciences, University of Milano-Bicocca, Piazza della Scienza 2, Milano 20126, Italy

*Corresponding author, Irina Borodina

Email: irbo@biosustain.dtu.dk

This PDF file includes:

Fig S1 to S7

Table S1 to S4

SI References

Other supporting materials for this manuscript include the following:

Dataset S1 to S4

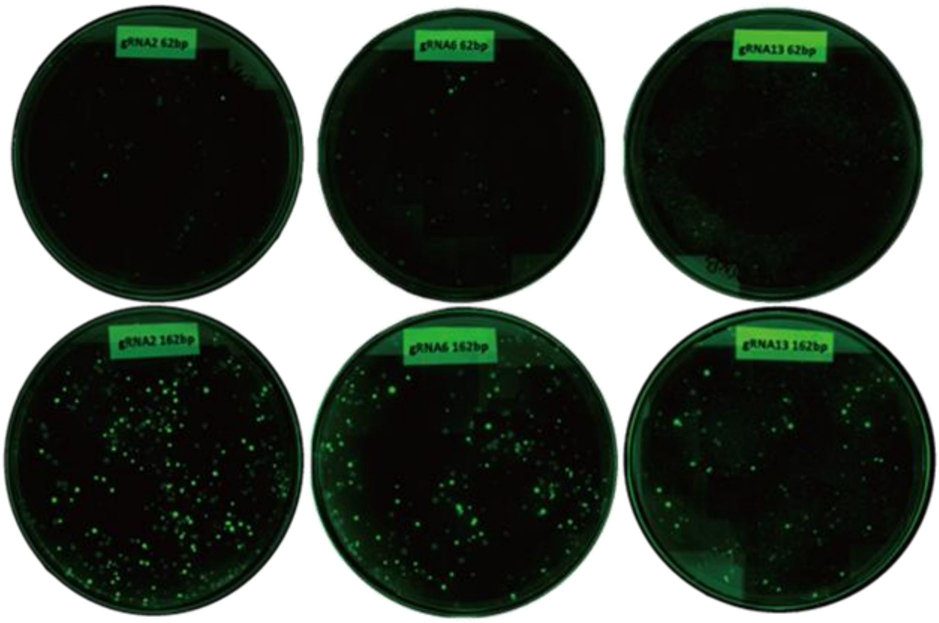

Fig S1. Fluorescent colony outcomes on transformation plates after swapping *PrURA3* with *PrTEF* to drive *mNG* expression using various designed sgRNAs.

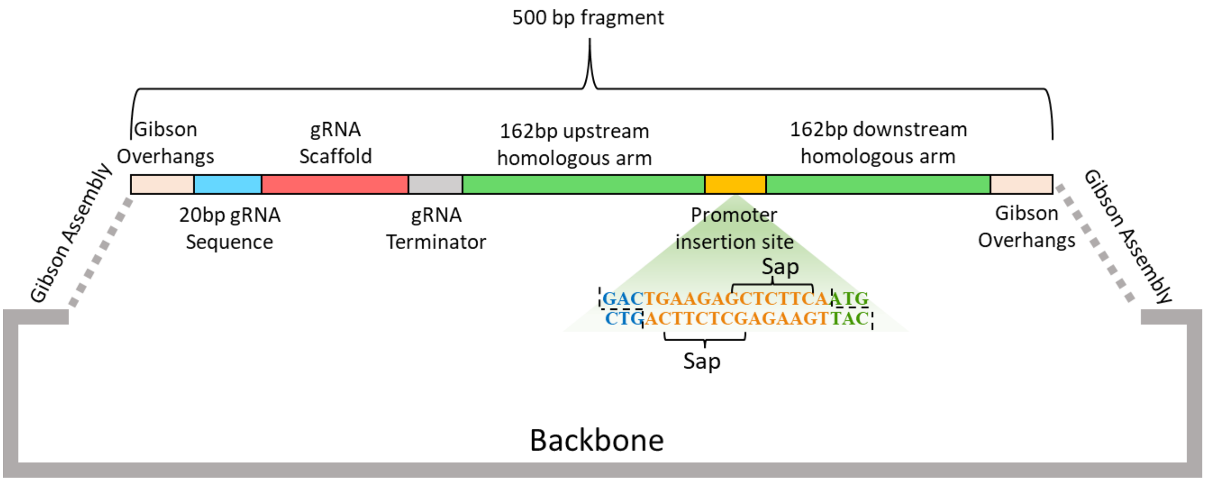

Fig S2. Plasmid design for the TUNE^{YALI} library

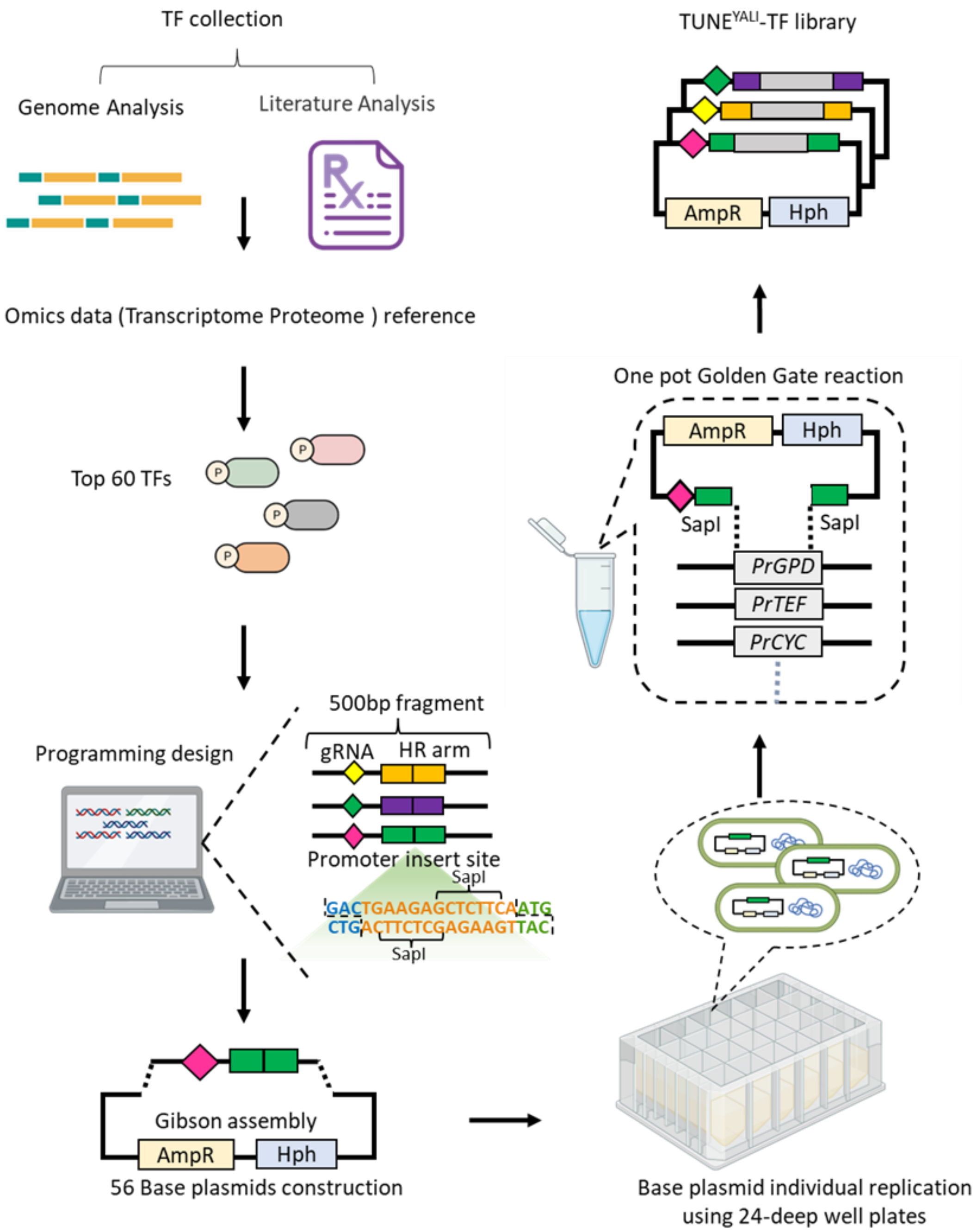

Fig S3. Schematic overview of the TUNE^{YALI}-TF library construction process. Key steps include TF collection and selection, HR arm design, base plasmid construction, and promoter integration via one-pot Golden Gate reactions. TF: transcription factor, HR: homologous recombination, AmpR: ampicillin, Hph: hygromycin

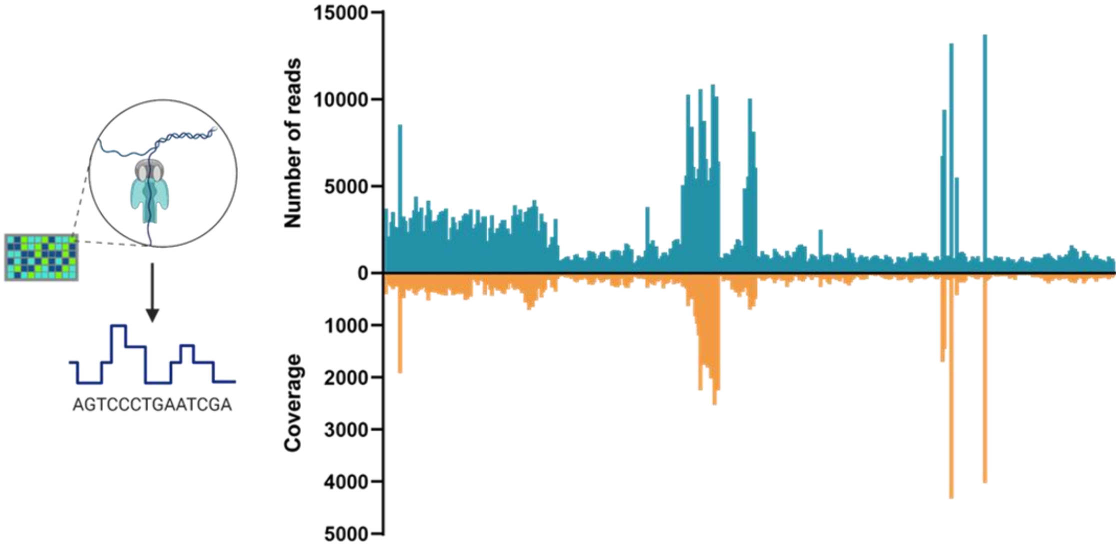

Fig S4. Nanopore sequencing results of the TUNE^{YALI}-TF library.

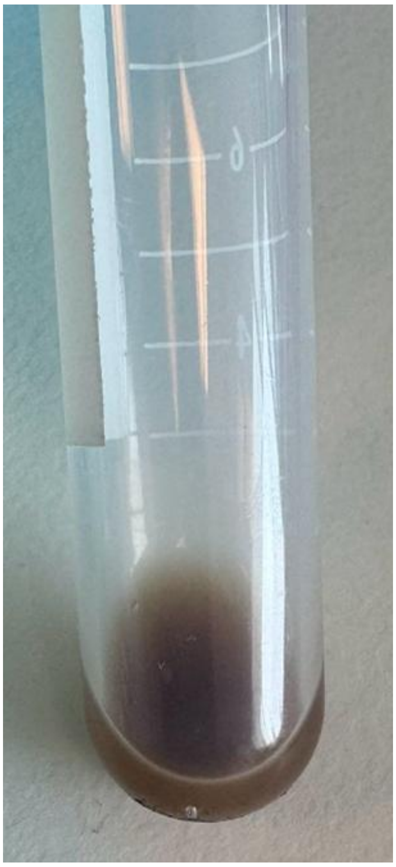

Fig S5. The culture medium of screened colonies with varying morphologies darkened.

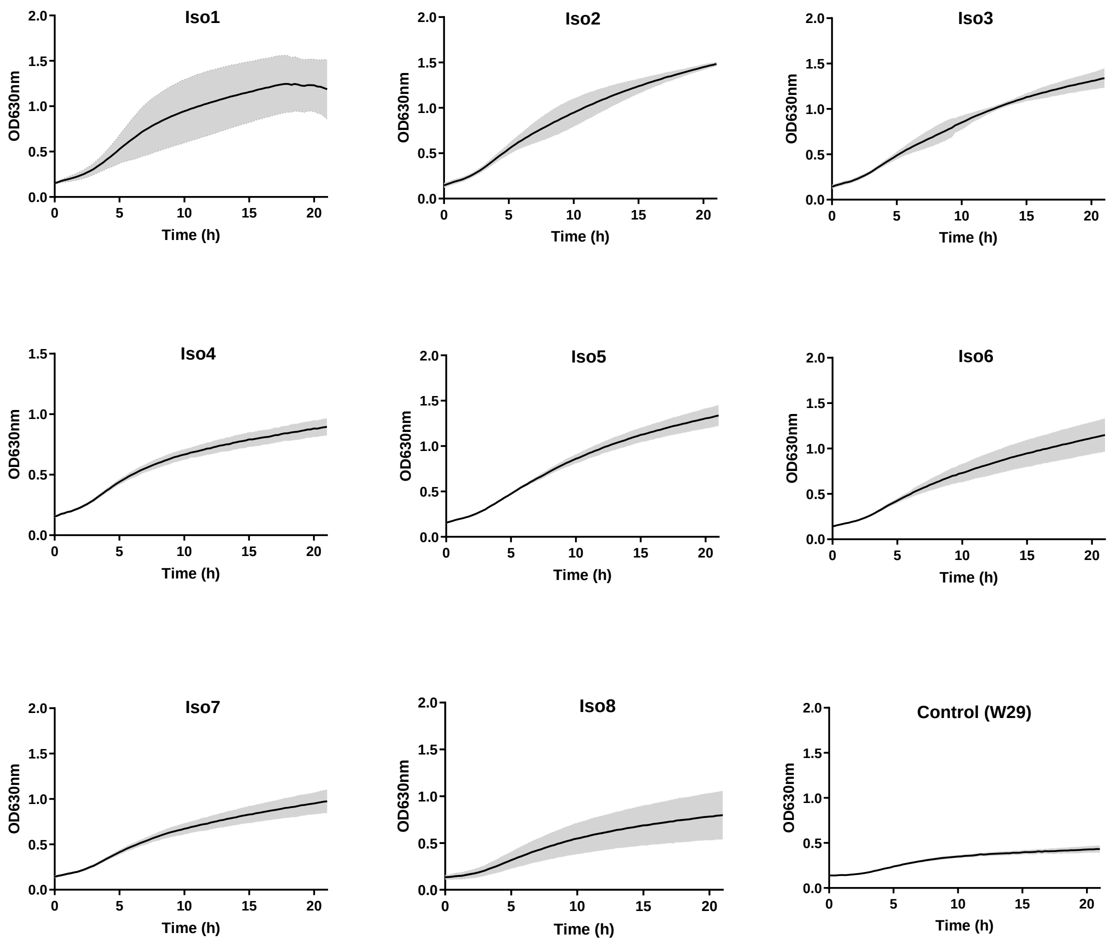

Strain	μmax (h-1)		Strain	μmax (h-1)	
	Ave	Stdev		Ave	Stdev
control	0.15	0.001	iso5	0.208	0.001
iso1	0.233	0.054	iso6	0.204	0.012
iso2	0.218	0.013	iso7	0.198	0.019
iso3	0.207	0.023	iso8	0.194	0.008
iso4	0.184	0			

Fig S6. Growth carves and maximum growth rate of *Y. lipolytica* isolates with enhanced temperature tolerance at 35 °C and control strain (W29).

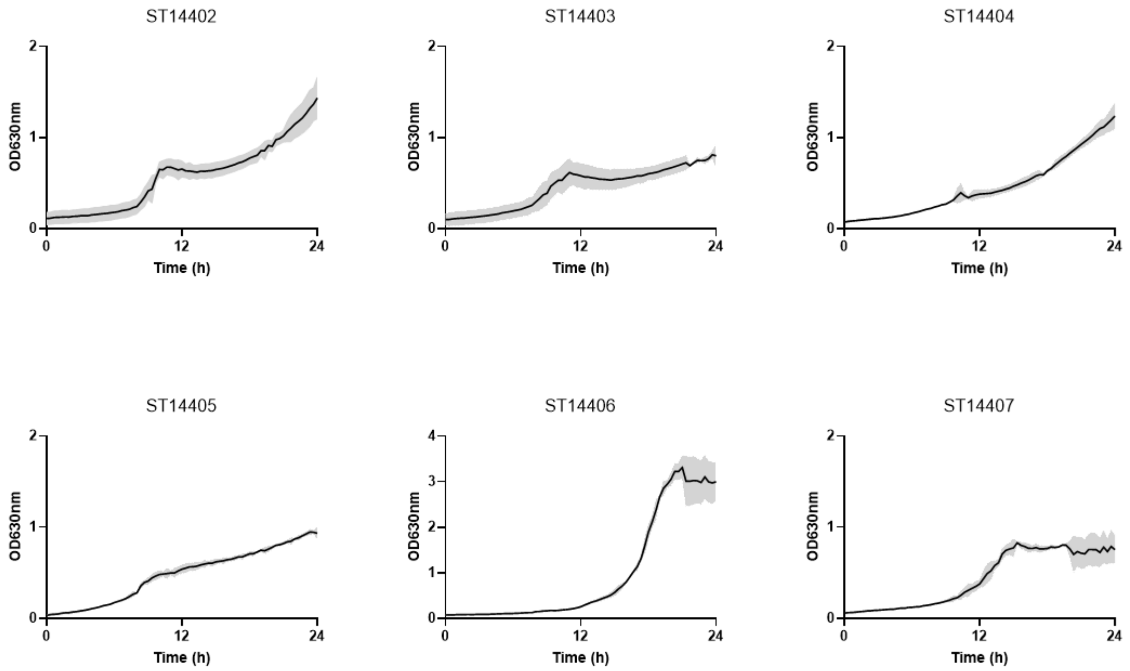

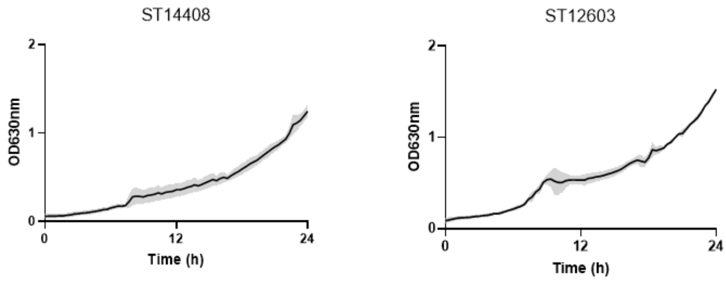

Strain	Lag time, h	μmax, /h	
		Average	Stdev
ST14402	8	0.297	0.081
ST14403	8	0.235	0.022
ST14404	11	0.156	0.024
ST14405	8	0.288	0.025
ST14406	14	0.138	0.049
ST14407	10	0.147	0.015
ST14408	10	0.192	0.050
ST12603	6	0.307	0.001

Strain	Genotype	Strain	Genotype
ST12603	Control strain	ST14402	*ΔPrYALI1_B06110g::PrTEF*
ST14403	*ΔPrYALI1_E16026g::PrDGA*	ST14404	*ΔPrYALI1_E32722g::PrDGA*
ST14405	*ΔPrYALI1_B06110g::PrTEF*<br>*ΔPrYALI1_E16026g::PrDGA*	ST14406	*ΔPrYALI1_E16026g::PrDGA*<br>*ΔPrYALI1_E32722g::PrDGA*
ST14407	*ΔPrYALI1_B06110g::PrTEF*<br>*ΔPrYALI1_E32722g::PrDGA*	ST14408	*ΔPrYALI1_B06110g::PrTEF*<br>*ΔPrYALI1_E16026g::PrDGA*<br>*ΔPrYALI1_E32722g::PrDGA*

Fig S7. Growth curves, lag time, and maximum growth rate of betanin-producing engineered strains (ST14402-ST14408) and control strain (ST12603).

Table S1 Selected high-throughput genome engineering toolboxes for *Y. lipolytica*

**Function**	**Uses and limitations**	**Reference and availability**
Genome-wide CRISPR-Cas9 gene deletion library	The method enables genome-wide gene deletions. Demonstrated for identification of essential genes and for identifying gene knock-out targets that improve lipid accumulation. Modifications are limited to the loss of gene function.	(1)
Genome-wide CRISPR-Cas9 gene deletion library. sgRNAs designed using deep learning	The method enables genome-wide gene deletions. The libraries were used for training the deep learning algorithm DeepGuide for improved sgRNA design.	(2)
Plasmid-based genomic DNA library harboring native promoters	The method introduces an additional copy of a genomic fragment on a replicating plasmid. Chromosomal DNA is not modified. Used for identification of genes involved in propionate tolerance.	(3)
EXPRESS^{YALI} method for combinatorial engineering	The method enables consecutive rounds of engineering, where up to three combinatorially assembled gene expression cassettes can be integrated into each yeast clone per round. The cassettes are integrated into distinct intergenic sites or an open reading frame of a target gene if a simultaneous gene knock-out is desired. Demonstrated for optimizing betanin production via six consecutive rounds of genome engineering and screening.	(4)<br>AddGene kit ID # 1000000245
TUNE^{YALI} method for regulating gene expression	The method enables modulation of gene expression by exchange of promoters. TUNE^{YALI}-TF library changes the expression of 56 transcription factors, which was used for improving betanin production, increasing thermotolerance, and eliminating pseudohyphal morphology. Libraries can be used iteratively.<br>The toolkit includes empty vectors for creating custom libraries targeting other genes.	This work.<br>AddGene library ID # 217744,<br>AddGene kit ID # 219866–219929

Table S2 Primers used in this study

Primer	Sequence	Description
PR-31973 FWD	ACCTAAGGAAACTGCTGAGTAATTTCGAGG	Common primers used to sequence gRNA
PR-31974 FWD	CAGTACCTCCCCTACCCCGACGGTATG	
PR-31975 REV	TATTCTCTAGAAAGTATAGGAACTTCACTTCATTT	
PR-31976 REV	TGGTAACCGGAACCGTCGACCATG	
PR-32075 FWD	GCTCTTCAGACTACCAACCACAGATTACGA	Amplify the promoter *PrPGM* with overhangs for the SapI restriction site
PR-32076 REV	GCTCTTCTCATTTTTGTATGTGTTTTGGTGATGT	
PR-34446 FWD	GCTCTTCAGACAGAGACCGGGTTGGCGGCG	Amplify the promoter *PrTEF* with overhangs for the SapI restriction site
PR-34447 REV	GCTCTTCTCATTTTGAATGATTCTTATACTCAG	
PR-34448 FWD	GCTCTTCAGACGACGCAGTAGGATGTCCTGC	Amplify the promoter *PrGPD* with overhangs for the SapI restriction site
PR-34449 REV	GCTCTTCTCATTGTTGATGTGTGTTTAATTC	
PR-34450 FWD	CAGATCAAGTAATCGTATCAATCGTTCCACGTCTTA	Sequencing the base plasmid of TUNE^{YALI}
PR-34451 FWD	AGATTACCTAATTTGGTAAGATATTAATTATG	
PR-34452 REV	AGAAAATAAATCTCGAAAATAATAAAGGGAA	
PR-34453 REV	CTATTCATCTATTATTTATGATTTTTTGT	
PR-32111 FWD	TGGACCGATAGCCGTATAGTCC	Yeast colony PCR to check *ΔURA3::mNG* in *Y. lipolytica*
PR-32112 REV	CGTACATGGGCTGGTTCTTGA	
PR-32186 FWD	CAGATAAGGTTCCGATAAAA	Sequence the gRNA plasmid targeting *PrURA3*
PR-32187 FWD	ATAGCCGTATAGTCCAGTCT	Sequence the gRNA plasmid targeting *PrURA3*
PR-32188 FWD	TTGACGTTCTTTCTAATTTG	Sequence the gRNA plasmid targeting *PrURA3*
PR-32191 FWD	GGAGGCTGTGGGTCTGACAG	Attach to the *Yarrowia* genome to check *ΔURA3::mNG* with 162bp HR arm
PR-32192 FWD	TCCAATTACCCCCCACAACA	Attach to the *Yarrowia* genome to check *ΔURA3::mNG* with 500bp HR arm

PR-32440 REV	GAAACCGGTACCCTTGACCTG	Attach to the *mNG*, used for yeast colony PCR to check *ΔURA3::mNG*
PR-32527 FWD	TCAAGGCGTGAACCACTGTTT	Yeast colony PCR to check *ΔPrURA3:: PrPGM* in *Y. lipolytica,* paired with PR-32440
PR-32528 FWD	AGCGCAACCCTATTCAGCG	Yeast colony PCR to check *ΔPrURA3:: PrPGM* in *Y. lipolytica,* paired with PR-32112
PR-32529 FWD	GGGCTGCACCCAACAATAAAT	Yeast colony PCR to check *ΔPrURA3:: PrFBA* in *Y. lipolytica,* paired with PR-32440
PR-32530 FWD	GCGAAAGCGCGTATGGATT	Yeast colony PCR to check *ΔPrURA3:: PrFBA* in *Y. lipolytica,* paired with PR-32112
PR-32537 FWD	GATAAAAAAACGCACCCACTGC	Yeast colony PCR to check *ΔPrURA3:: PrDGA* in *Y. lipolytica,* paired with PR-32440
PR-32538 FWD	GCTCCTCGAAACCGACTCCT	Yeast colony PCR to check *ΔPrURA3:: PrDGA* in *Y. lipolytica,* paired with PR-32112
PR-32539 FWD	GATAGAGTGTGGGTCACGCAGA	Yeast colony PCR to check *ΔPrURA3:: PrCYC*in *Y. lipolytica,* paired with PR-32440
PR-32540 FWD	GTTTGGGATTGGGGAATTGG	Yeast colony PCR to check *ΔPrURA3:: PrCYC* in *Y. lipolytica,* paired with PR-32112
PR-32541 FWD	CCCCCAAAACCTCCACTGTAC	Yeast colony PCR to check *ΔPrURA3:: PrEXP* in *Y. lipolytica,* paired with PR-32440

PR-32542 FWD	AACGCCTCTCCGACTCCCT	Yeast colony PCR to check *ΔPrURA3:: PrEXP* in *Y. lipolytica,* paired with PR-32112
PR-32543 FWD	GGTTCCCAAGGAAAAGGTCG	Negative control of Yeast colony PCR to check *the replacement of PrURA3*, paired with PR-32440
PR-32597 FWD	AGTTCAGGCTTTTTACCCATTGCTGTAGATATGTCTTGTGTGTAAGGGGG	Amplify the backbone of 11863 plasmid, Gibson assembly with the *hphMX*
PR-32598 REV	AGAAGGTTTTGGGACGCTCGAGCAGGCTTGGAGGCGACGT	
PR-32599 FWD	CACAAGACATATCTACAGCAATGGGTAAAAAGCCTGAACTCACC	Amplify the gene *hphMX*, Gibson assembly with the backbone of 11863 plasmid
PR-32600 REV	ACGTCGCCTCCAAGCCTGCTCGAGCGTCCCAAAACCTTCT	
PR-32601 FWD	CGGCGTCCACTTGCACAAAC	Sequence the gene *hphMX* after Gibson assembly with the backbone of 11863 plasmid
PR-32602 REV	CCTGCACATGTCACCTGTCT	
PR-32603 REV	CCTGTTCAAGGGCAAAGTCTAGA	
PR-32622 FWD	GCTCTTCAGACTTGACCAACCTTGT	Amplify the promoter *PrACT* with overhangs for the SapI restriction site
PR-32623 REV	GCTCTTCTCATTTTTAATTTGTGTGGTTG	
PR-32624 FWD	GCTCTTCAGACTTAGTTGACTGGAAAA	Amplify the promoter *PrTPI* with overhangs for the SapI restriction site
PR-32625 REV	GCTCTTCTCATTTTGAATGTAGTTGTGTT	
PR-32696 FWD	TCTATACGCCTTTTGACGCCA	Yeast colony PCR to check *ΔPrURA3:: PrTPI* in *Y. lipolytica,* paired with PR-32440
PR-32697 FWD	ACCGAGAGGACCCCAAAAAG	Yeast colony PCR to check *ΔPrURA3:: PrACTI* in *Y. lipolytica,* paired with PR-32440
PR-34229 FWD	TGTCTATGCCATGTCCATGATCATGTAATTAGTTATGTCACGC	Amplify the backbone of $TUNE^{YALI}$ plasmid to Gibson assembly with the gene *natMX*
PR-34230 REV	TCATCCAAAGTAGTACCCATTGCTGTAGATATGTCTTGTGTG	
PR-34231 FWD	CACAAGACATATCTACAGCAATGGGTACTACTTTGGATGATAC	Amplify the gene *natMX* to Gibson assembly with the backbone of $TUNE^{YALI}$ plasmid
PR-34232 REV	TGACATAACTAATTACATGATCATGGACATGGCATAGACA	

PR-34259 FWD	ACGTGTGTAGAGGAGTCGGTTTCG	Yeast colony PCR to verify that the native promoter of TF23 was replaced by promoter *PrDGA*
PR-34252 REV	TGGCAAGAAGTGGCATCTTCA	
PR-34261 FWD	ACACTCCCTTTCCATTCCC	Yeast colony PCR to verify that the native promoter of TF14 was replaced by promoter *PrDGA*
PR-34254 REV	ACATGCCACCCATGTTCA	
PR-34260 FWD	ACTTGCCGTTAAGGGCGT	Yeast colony PCR to verify that the native promoter of TF56 was replaced by promoter *PrTEF*
PR-34256 REV	CATCTGCCATTATGTCAACCG	
PR-32126 REV	TTTGGTGGTGAAGAGGAGAC	Amplify 500bp upstream of *URA3* in *Y. lipolytica*
PR-32128 FWD	AAGTTGGTGAGAAATGGACC	
PR-32129 REV	AACTTTTCGATGTCCCGTTT	Amplify 500bp downstream of *URA3* in *Y. lipolytica*
PR-32127 FWD	AGGTTAGACTATGGATATGTAATTTAACTGTG	
PR-32130 FWD	AAACGGGACATCGAAAAGTTATCGCGTGCATTCGCGGCCG	Amplify the backbone of the gRNA plasmid to construct the plasmid pCfb12570 by Gibson assembly
PR-32131 REV	GGTCCATTTCTCACCAACTTACCGTACCCACACAAAAAAAGCACCACCG A	
PR-32132 FWD	GTCCAATGGGGCATGTTGTTATCGCGTGCATTCGCGGCCG	Amplify the backbone of the gRNA plasmid to construct the plasmid pCfb12569 by Gibson assembly
PR-32133 REV	TCCAAATTAGAAAGAACGTCACCGTACCCACACAAAAAAAGCACCACC GA	
PR-32113 FWD	AGGTTAGACTATGGATATGTAATTTAACTGTGTATATAGAGAGCGTGC AAGTATGGAGCGCTTGTTCAGCTTGTATGATGGTCAGACGACC	Amplify 162bp downstream of *URA3* in *Y. lipolytica*
PR-32114 REV	AACAACATGCCCCATTGGACAGATCATGCGGATACACAGGTTGTGCA GTATCATACATACTCGATCAGACAGGTCGTCTGACCATCATACA	
PR-32134 FWD	GACGTTCTTTCTAATTTGGACCGATAGCCGTATAGTCCAGTCTAT CTATAAGTTCAACTAACTCGTAACTATTACCATAACATATACTTCA	Amplify 162bp downstream of *URA3* in *Y. lipolytica*
PR-32135 REV	TTTGGTGGTGAAGAGGAGACTGAAATAAATTTAGTCTGCAGAA CTTTTTATCGGAACCTTATCTGGGGCAGTGAAGTATATGTTATGGTAA	

Table S3 Plasmids used in this study

Name	Description	Parental plasmid	Reference
pCfb11606	The plasmid containing gRNA unit and 62bp HR arm to knock *URA3* out with Nat selection maker		This work
pCfb12563	The plasmid containing gRNA unit, 62bp HR arm and *mNG* for *ΔURA3::mNG*	Cfb11606	This work
pCfb12561	*PrPGM* with the overhangs for SapI restriction site was cloned into the Blunt TOPO® vector (Invitrogen™, 450245)		This work
pCfb12562	*PrGPD* with the overhangs for SapI restriction site was cloned into the Blunt TOPO® vectors(Invitrogen™, 450245)		This work
pCfb13186	*PrTEF* with the overhangs for SapI restriction site was cloned into the Blunt TOPO® vectors(Invitrogen™, 450245)		This work
pCfb12565	*PrFBA* with the overhangs for SapI restriction site was cloned into the Blunt TOPO® vector (Invitrogen™, 450245)		This work
pCfb12566	*PrDGA* with the overhangs for SapI restriction site was cloned into the Blunt TOPO® vector (Invitrogen™, 450245)		This work
pCfb12567	*PrCYC* with the overhangs for SapI restriction site was cloned into the Blunt TOPO® vector (Invitrogen™, 450245)		This work
pCfb12620	*PrTPI* with the overhangs for SapI restriction site was cloned into the Blunt TOPO® vector (Invitrogen™, 450245)		This work
pCfb12622	*PrACT* with the overhangs for SapI restriction site was cloned into the Blunt TOPO® vector (Invitrogen™, 450245)		This work
pCfb12569	The plasmid containing gRNA unit, 162bp HR arm and *mNG* for *ΔURA3::mNG*	Cfb11606	This work
pCfb12570	The plasmid containing gRNA unit, 500bp HR arm and *mNG* for *ΔURA3::mNG*	Cfb11606	This work
pCfb12616	This plasmid was used for knocking *PrURA3* out and promoter insertion with gRNA2 and 162bp HR arm		

pCfb12617	This plasmid was used for *PrURA3* and promoter insertion with gRNA6 and 162bp HR arm		
pCfb13189	This plasmid was used for knocking *PrURA3* out and promoter insertion with gRNA2 and 62bp HR arm		
pCfb13190	This plasmid was used for knocking *PrURA3* out and promoter insertion with gRNA6 and 62bp HR arm		
pCfb12579	This plasmid was used for *ΔPrURA3::PrTEF* with gRNA2 and 62bp HR arm	pCfb13189<br>pCfb13186	This work
pCfb12580	This plasmid was used for *ΔPrURA3::PrTEF* with gRNA2 and 162bp HR arm	pCfb12616<br>pCfb13186	This work
pCfb13187	This plasmid was used for *ΔPrURA3::PrTEF* with gRNA6 and 62bp HR arm	pCfb13190<br>pCfb13186	This work
pCfb12582	This plasmid was used for *ΔPrURA3::PrTEF* with gRNA6 and 162bp HR arm	pCfb12617<br>pCfb13186	This work
pCfb12584	This plasmid was used for *ΔPrURA3::PrTEF* with gRNA13 and 62bp HR arm	pCfb13186	This work
pCfb12585	This plasmid was used for *ΔPrURA3::PrTEF* with gRNA13 and 162bp HR arm	pCfb13186	This work
pCfb12575	This plasmid was used for *ΔPrURA3::PrDGA* with gRNA2 and 62bp HR arm	pCfb13189<br>pCfb12566	This work
pCfb12593	This plasmid was used for *ΔPrURA3:: PrDGA* with gRNA2 and 162bp HR arm	pCfb12616<br>pCfb12566	This work
pCfb12588	This plasmid was used for *ΔPrURA3::PrFBA* with gRNA2 and 62bp HR arm	pCfb13189<br>pCfb12565	This work
pCfb12589	This plasmid was used for *ΔPrURA3::PrFBA* with gRNA2 and 162bp HR arm	pCfb12616<br>pCfb12565	This work
pCfb12594	This plasmid was used for *ΔPrURA3::PrPGM* with gRNA2 and 62bp HR arm	pCfb13189<br>pCfb12561	This work
pCfb12578	This plasmid was used for *ΔPrURA3::PrPGM* with gRNA2 and 162bp HR arm	pCfb12616<br>pCfb12561	This work
pCfb12610	This plasmid was used for *ΔPrURA3::PrCYC* with gRNA2 and 62bp HR arm	pCfb13189	This work

		pCfb12567	
pCfb12611	This plasmid was used for *ΔPrURA3::PrCYC* with gRNA2 and 162bp HR arm	pCfb12616<br>pCfb12567	This work
pCfb13188	This plasmid was used for *ΔPrURA3::PrGPD* with gRNA2 and 62bp HR arm	pCfb13189<br>pCfb12562	This work
pCfb12613	This plasmid was used for *ΔPrURA3::PrGPD* with gRNA2 and 162bp HR arm	pCfb12616<br>pCfb12562	This work
pCfb12614	This plasmid was used for *ΔPrURA3::PrTPI* with gRNA2 and 162bp HR arm	pCfb13189<br>pCfb12620	This work
pCfb12615	This plasmid was used for *ΔPrURA3::PrACT* with gRNA2 and 162bp HR arm	pCfb12616<br>pCfb12622	This work
pCfb12618	gRNA plasmid with the selection maker hygromycin	pCfb11606	This work
pCfb13191	The base plasmid of $TUNE^{YALI}$ for TF01	pCfb12618	This work
pCfb13192	The base plasmid of $TUNE^{YALI}$ for TF02	pCfb12618	This work
pCfb13193	The base plasmid of $TUNE^{YALI}$ for TF03	pCfb12618	This work
pCfb13194	The base plasmid of $TUNE^{YALI}$ for TF04	pCfb12618	This work
pCfb13195	The base plasmid of $TUNE^{YALI}$ forTF05	pCfb12618	This work
pCfb13196	The base plasmid of $TUNE^{YALI}$ forTF06	pCfb12618	This work
pCfb13197	The base plasmid of $TUNE^{YALI}$ forTF07	pCfb12618	This work
pCfb13198	The base plasmid of $TUNE^{YALI}$ forTF08	pCfb12618	This work
pCfb13199	The base plasmid of $TUNE^{YALI}$ forTF09	pCfb12618	This work
pCfb13200	The base plasmid of $TUNE^{YALI}$ forTF10	pCfb12618	This work
pCfb13201	The base plasmid of $TUNE^{YALI}$ forTF11	pCfb12618	This work
pCfb13202	The base plasmid of $TUNE^{YALI}$ forTF12	pCfb12618	This work
pCfb13203	The base plasmid of $TUNE^{YALI}$ forTF13	pCfb12618	This work
pCfb13204	The base plasmid of $TUNE^{YALI}$ for TF14	pCfb12618	This work
pCfb13205	The base plasmid of $TUNE^{YALI}$ for TF15	pCfb12618	This work
pCfb13206	The base plasmid of $TUNE^{YALI}$ for TF16	pCfb12618	This work
pCfb13207	The base plasmid of $TUNE^{YALI}$ for TF17	pCfb12618	This work

pCfb13208	The base plasmid of $TUNE^{YALI}$ for TF18	pCfb12618	This work
pCfb13209	The base plasmid of $TUNE^{YALI}$ for TF19	pCfb12618	This work
pCfb13210	The base plasmid of $TUNE^{YALI}$ for TF20	pCfb12618	This work
pCfb13211	The base plasmid of $TUNE^{YALI}$ for TF21	pCfb12618	This work
pCfb13212	The base plasmid of $TUNE^{YALI}$ for TF22	pCfb12618	This work
pCfb13213	The base plasmid of $TUNE^{YALI}$ for TF23	pCfb12618	This work
pCfb13214	The base plasmid of $TUNE^{YALI}$ for TF24	pCfb12618	This work
pCfb13215	The base plasmid of $TUNE^{YALI}$ for TF25	pCfb12618	This work
pCfb13216	The base plasmid of $TUNE^{YALI}$ for TF26	pCfb12618	This work
pCfb13217	The base plasmid of $TUNE^{YALI}$ for TF27	pCfb12618	This work
pCfb13218	The base plasmid of $TUNE^{YALI}$ for TF28	pCfb12618	This work
pCfb13219	The base plasmid of $TUNE^{YALI}$ for TF29	pCfb12618	This work
pCfb13220	The base plasmid of $TUNE^{YALI}$ for TF30	pCfb12618	This work
pCfb13221	The base plasmid of $TUNE^{YALI}$ for TF31	pCfb12618	This work
pCfb13222	The base plasmid of $TUNE^{YALI}$ for TF32	pCfb12618	This work
pCfb13223	The base plasmid of $TUNE^{YALI}$ for TF33	pCfb12618	This work
pCfb13224	The base plasmid of $TUNE^{YALI}$ for TF34	pCfb12618	This work
pCfb13225	The base plasmid of $TUNE^{YALI}$ for TF35	pCfb12618	This work
pCfb13226	The base plasmid of $TUNE^{YALI}$ for TF36	pCfb12618	This work
pCfb13227	The base plasmid of $TUNE^{YALI}$ for TF37	pCfb12618	This work
pCfb13228	The base plasmid of $TUNE^{YALI}$ for TF38	pCfb12618	This work
pCfb13229	The base plasmid of $TUNE^{YALI}$ for TF39	pCfb12618	This work
pCfb13231	The base plasmid of $TUNE^{YALI}$ for TF41	pCfb12618	This work
pCfb13233	The base plasmid of $TUNE^{YALI}$ for TF43	pCfb12618	This work
pCfb13234	The base plasmid of $TUNE^{YALI}$ for TF44	pCfb12618	This work
pCfb13235	The base plasmid of $TUNE^{YALI}$ for TF45	pCfb12618	This work
pCfb13236	The base plasmid of $TUNE^{YALI}$ for TF46	pCfb12618	This work
pCfb13237	The base plasmid of $TUNE^{YALI}$ for TF47	pCfb12618	This work
pCfb13238	The base plasmid of $TUNE^{YALI}$ for TF48	pCfb12618	This work

pCfb13239	The base plasmid of TUNE^{YALI} for TF49	pCfb12618	This work
pCfb13240	The base plasmid of TUNE^{YALI} for TF50	pCfb12618	This work
pCfb13242	The base plasmid of TUNE^{YALI} for TF52	pCfb12618	This work
pCfb13244	The base plasmid of TUNE^{YALI} for TF54	pCfb12618	This work
pCfb13245	The base plasmid of TUNE^{YALI} for TF55	pCfb12618	This work
pCfb13246	The base plasmid of TUNE^{YALI} for TF56	pCfb12618	This work
pCfb13247	The base plasmid of TUNE^{YALI} for TF57	pCfb12618	This work
pCfb13248	The base plasmid of TUNE^{YALI} for TF58	pCfb12618	This work
pCfb13249	The base plasmid of TUNE^{YALI} for TF59	pCfb12618	This work
pCfb13250	The base plasmid of TUNE^{YALI} for TF60	pCfb12618	This work
pCfB13166	The plasmid with the selection maker hygromycin was used for *ΔPrYALI1_B06110g* (TF56)*::PrTEF*	pCfb13246<br>pCfb13186	This work
pCfB13169	The plasmid with the selection maker hygromycin was used for *ΔPrΔPrYALI1_E16026g* (TF14)*::PrDGA*	pCfb12618<br>pCfb12566	This work
pCfB13170	The plasmid with the selection maker hygromycin was used for *ΔPrYALI1_E32722g* (TF23)*::PrDGA*	pCfb13213<br>pCfb12566	This work
pCfB13173	The plasmid with the selection maker nourseothricin was used for *ΔPrYALI1_E32722g (TF23)::PrDGA*	pCfB13170	This work
pCfB13175	The plasmid with the selection maker nourseothricin was used for *ΔPrYALI1_B06110g (TF56)::PrTEF*	pCfB13166	This work

44

Table S4 strains used in this study

**Strain No.**	**Genotype**	**Parental strains**	**Source**
ST6512	*MATa Δku70::PrTEF1-cas9-TTef12::PrGPD-dsdAMX-TLip2*<br>*(MATa Δku70::SpCas9-EcDsdAMX4*)	W29 (Y-63746)	(5)
ST14141	*MATa Δku70::SpCas9-EcDsdAMX4,*<br>*ΔURA3::mNG*	ST6512	This study
ST14142	*MATa Δku70::SpCas9-EcDsdAMX4,*<br>*ΔURA3::mNG*<br>*ΔPrURA3*	ST14141	This study
ST14143	*MATa Δku70::SpCas9-EcDsdAMX4,*<br>*ΔURA3::mNG*<br>*ΔPrURA3::PrTEF*	ST14141	This study
ST14144	*MATa Δku70::SpCas9-EcDsdAMX4,*<br>*ΔURA3::mNG*<br>*ΔPrURA3::PrFBA*	ST14141	This study
ST14145	*MATa Δku70::SpCas9-EcDsdAMX4,*<br>*ΔURA3::mNG*<br>*ΔPrURA3::PrCYC*	ST14141	This study
ST14146	*MATa Δku70::SpCas9-EcDsdAMX4,*<br>*ΔURA3::mNG*<br>*ΔPrURA3::PrDGA*	ST14141	This study
ST14147	*MATa Δku70::SpCas9-EcDsdAMX4,*<br>*ΔURA3::mNG*<br>*ΔPrURA3::PrPGM*	ST14141	This study
ST14148	*MATa Δku70::SpCas9-EcDsdAMX4,*<br>*ΔURA3::mNG*<br>*ΔPrURA3::PrGPD*	ST14141	This study
ST14149	*MATa Δku70::SpCas9-EcDsdAMX4,*<br>*ΔURA3::mNG*	ST14141	This study

	ΔPrURA3::PrACT		
ST14150	*MATa Δku70::SpCas9-EcDsdAMX4,*<br>*ΔURA3::mNG*<br>*ΔPrURA3::PrTPI*	ST14141	This study
ST12603	*MATa Δku70::SpCas9-EcDsdAMX4,*<br>*Pex20-YlARO7^{G139S}-PrGPD-PrTEFin-YlARO4^{K221L}-tLip2*<br>*3x TPex20-BvGT2-PrTEFin*<br>*3x TPex20-MjDOD-PrGPD-PrTEFin-EvTYH-TLip2*<br>*Δ4hppd*	ST6512	(6)
ST14402	*MATa Δku70::SpCas9-EcDsdAMX4,*<br>*Pex20-YlARO7^{G139S}-PrGPD-PrTEFin-YlARO4^{K221L}-tLip2*<br>*3x TPex20-BvGT2-PrTEFin*<br>*3x TPex20-MjDOD-PrGPD-PrTEFin-EvTYH-TLip2*<br>*Δ4hppd*<br>*ΔPrYALI1_B06110g* (TF56)*::PrTEF*	ST12603	This study
ST14403	*MATa Δku70::SpCas9-EcDsdAMX4,*<br>*Pex20-YlARO7^{G139S}-PrGPD-PrTEFin-YlARO4^{K221L}-tLip2*<br>*3x TPex20-BvGT2-PrTEFin*<br>*3x TPex20-MjDOD-PrGPD-PrTEFin-EvTYH-TLip2*<br>*Δ4hppd*<br>*ΔPrYALI1_E16026g* (TF14)*::PrDGA*	ST12603	This study
ST14404	*MATa Δku70::SpCas9-EcDsdAMX4,*<br>*Pex20-YlARO7^{G139S}-PrGPD-PrTEFin-YlARO4^{K221L}-tLip2*<br>*3x TPex20-BvGT2-PrTEFin*<br>*3x TPex20-MjDOD-PrGPD-PrTEFin-EvTYH-TLip2*<br>*Δ4hppd*<br>*ΔPrYALI1_E32722g* (TF23)*::PrDGA*	ST12603	This study

ST14405	*MATa Δku70::SpCas9-EcDsdAMX4,*<br>*Pex20-YlARO7^{G139S}-PrGPD-PrTEFin-YlARO4^{K221L}-tLip2*<br>*3x TPex20-BvGT2-PrTEFin*<br>*3x TPex20-MjDOD-PrGPD-PrTEFin-EvTYH-TLip2*<br>*Δ4hppd*<br>*ΔPrYALI1_B06110g* (TF56)*::PrTEF*<br>*ΔPrYALI1_E16026g* (TF14)*::PrDGA*	ST12603	This study
ST14406	*MATa Δku70::SpCas9-EcDsdAMX4,*<br>*Pex20-YlARO7^{G139S}-PrGPD-PrTEFin-YlARO4^{K221L}-tLip2*<br>*3x TPex20-BvGT2-PrTEFin*<br>*3x TPex20-MjDOD-PrGPD-PrTEFin-EvTYH-TLip2*<br>*Δ4hppd*<br>*ΔPrYALI1_E16026g* (TF14)*::PrDGA*<br>*ΔPrYALI1_E32722g* (TF23)*::PrDGA*	ST12603	This study
ST14407	*MATa Δku70::SpCas9-EcDsdAMX4,*<br>*Pex20-YlARO7^{G139S}-PrGPD-PrTEFin-YlARO4^{K221L}-tLip2*<br>*3x TPex20-BvGT2-PrTEFin*<br>*3x TPex20-MjDOD-PrGPD-PrTEFin-EvTYH-TLip2*<br>*Δ4hppd*<br>*ΔPrYALI1_B06110g* (TF56)*::PrTEF*<br>*ΔPrYALI1_E32722g* (TF23)*::PrDGA*	ST12603	This study
ST14408	*MATa Δku70::SpCas9-EcDsdAMX4,*<br>*Pex20-YlARO7^{G139S}-PrGPD-PrTEFin-YlARO4^{K221L}-tLip2*<br>*3x TPex20-BvGT2-PrTEFin*<br>*3x TPex20-MjDOD-PrGPD-PrTEFin-EvTYH-TLip2*<br>*Δ4hppd*<br>*ΔPrYALI1_B06110g* (TF56)*::PrTEF*<br>*ΔPrYALI1_E32722g* (TF23)*::PrDGA*<br>*ΔPrYALI1_E16026g* (TF14)*::PrDGA*	ST12603	This study

Dataset S1 (separate file). The transcription factor collection, the 60 top-selected transcription factors, and the 56 gRNA sequences corresponding to 56 final transcription factors in the TUNE^{YALI} kit.

Dataset S2 (separate file). Nanopore sequence reads and depths.

Dataset S3 (separate file). The OD_{600} and betanin absorbance of betanin-producing isolates selected using the PIXL colony picker.

Dataset S4 (separate file). The OD_{600} and betanin absorbance of betanin-producing isolates selected using the PIXL colony picker through iterative application of the TUNE^{YALI}-TF library.

SI References

1. C. Schwartz *et al.*, Validating genome-wide CRISPR-Cas9 function improves screening in the oleaginous yeast Yarrowia lipolytica. *Metabolic engineering* **55**, 102-110 (2019).
2. N. R. Robertson *et al.*, Optimized genome-wide CRISPR screening enables rapid engineering of growth-based phenotypes in Yarrowia lipolytica. *Metabolic Engineering* **86**, 55-65 (2024).
3. Y. K. Park, J. M. Nicaud, Screening a genomic library for genes involved in propionate tolerance in Yarrowia lipolytica. *Yeast* **37**, 131-140 (2020).
4. W. Jiang *et al.*, Combinatorial iterative method for metabolic engineering of Yarrowia lipolytica: Application for betanin biosynthesis. *Metabolic Engineering* **86**, 78-88 (2024).
5. E. R. Marella *et al.*, A single-host fermentation process for the production of flavor lactones from non-hydroxylated fatty acids. *Metabolic engineering* **61**, 427-436 (2020).
6. P. T. Thomsen *et al.*, Beet red food colourant can be produced more sustainably with engineered Yarrowia lipolytica. *Nature Microbiology* **8**, 2290-2303 (2023).
